# Perturbation of the P-Body Component Mov10 Inhibits HIV-1 Infectivity

**DOI:** 10.1371/journal.pone.0009081

**Published:** 2010-02-05

**Authors:** Vyacheslav Furtak, Alok Mulky, Stephen A. Rawlings, Lina Kozhaya, KyeongEun Lee, Vineet N. KewalRamani, Derya Unutmaz

**Affiliations:** 1 Department of Microbiology, New York University School of Medicine, New York, New York, United States of America; 2 Department of Pathology, New York University School of Medicine, New York, New York, United States of America; 3 HIV Drug Resistance Program, National Cancer Institute, Frederick, Maryland, United States of America; Sanofi-Aventis, United States of America

## Abstract

Exogenous retroviruses are obligate cellular parasites that co-opt a number of host proteins and functions to enable their replication and spread. Several host factors that restrict HIV and other retroviral infections have also recently been described. Here we demonstrate that Mov10, a protein associated with P-bodies that has a putative RNA-helicase domain, when overexpressed in cells can inhibit the production of infectious retroviruses. Interestingly, reducing the endogenous Mov10 levels in virus-producing cells through siRNA treatment also modestly suppresses HIV infectivity. The actions of Mov10 are not limited to HIV, however, as ectopic expression of Mov10 restricts the production of other lentiviruses as well as the gammaretrovirus, murine leukemia virus. We found that HIV produced in the presence of high levels of Mov10 is restricted at the pre-reverse transcription stage in target cells. Finally, we show that either helicase mutation or truncation of the C-terminal half of Mov10, where a putative RNA-helicase domain is located, maintained most of its HIV inhibition; whereas removing the N-terminal half of Mov10 completely abolished its activity on HIV. Together these results suggest that Mov10 could be required during the lentiviral lifecycle and that its perturbation disrupts generation of infectious viral particles. Because Mov10 is implicated as part of the P-body complex, these findings point to the potential role of cytoplasmic RNA processing machinery in infectious retroviral production.

## Introduction

The replication of retroviruses within target cells requires the participation of host factors at every step of the virus lifecycle. Indeed, genetic screens have suggested hundreds of host factors to contribute to HIV-1 replication [1]. As a consequence, hosts have developed potent retroviral restrictive proteins, which act as an intrinsic defense mechanism [2], [3]. Among the most prominent of this group are the APOBEC3 proteins, which manifest a potent cellular defense mechanism that has expanded in the primate family to prevent infection by viruses that require the production of ssDNA as part of their lifecycle [4]. This model of an antiviral countermeasure has been of particular importance in the quest to better understand the interaction between HIV-1 and various host proteins. Though APOBEC3G was initially identified as an inhibitor of HIV-1 replication in the absence of *vif* [5], it has since been suggested that APOBEC3G exerts a more nuanced role inside the cell and during the replication cycle of HIV-1 [6].

Recent evidence suggests a complex interplay between APOBEC3G and the cytoplasmic foci of proteins, referred to as P-bodies, which are thought to be involved in variety of RNA processing functions [7], [8]. One of the components of P-body complexes is a protein called Mov10, which was found to be associated with APOBEC3G in large complexes [6], [9]. Mov10 was first identified in screens that examined failure of infectious Moloney murine leukemia virus (MLV) production in mice [10]. Subsequent sequence analysis revealed seven consensus sequences of RNA helicases at the C-terminal end of the protein [11]. Recent evidence implicates Mov10 as a host factor required for hepatitis D virus replication [12]. In this study, it was shown that knockdown of Mov10 in host cells by siRNA significantly reduced hepatitis D viral replication, but not hepatitis D antigen production. [12].

Similar to previous reports, we found that Mov10 was the predominant protein associated with APOBEC3G in large protein complexes. We therefore examined the role of Mov10 in the retroviral lifecycle and discovered that the perturbation of Mov10 levels in producer cells greatly reduces the infectivity of HIV-1 and other retroviruses. Furthermore, we found that HIV-1 produced in the presence of high levels of Mov10 is restricted in infection of target cells either prior to or at the initiation of reverse transcription. Structure function analysis of Mov10 suggested that this potent activity on infectivity of HIV-1 is not dependent on its putative RNA-helicase domain.

## Results

### Overexpression of Mov10 Reduces the Specific Infectivity of HIV-1

**Figure 1:**
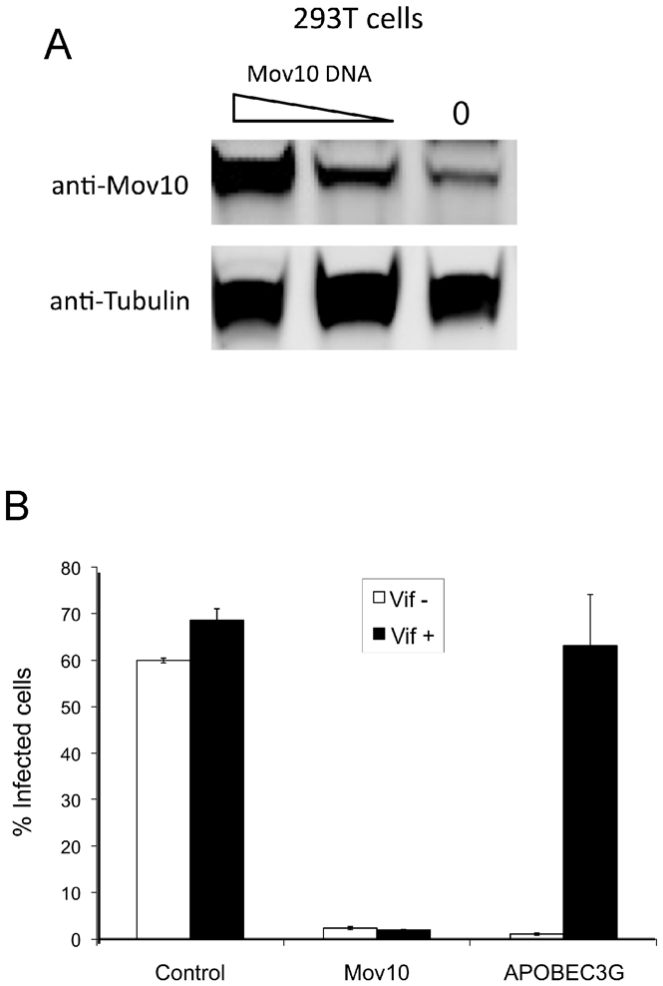
Overexpression of Mov10 decreases HIV-1 infectivity. (**A**) 293T cells were transfected with different amounts of Mov10 plasmid, and the expression of Mov10 was determined by Western blot. (**B**) 293T cells were transfected with 0.5 µg of either pCMV6-XL5 plasmid (control), Mov10 or APOBEC3G in the presence or absence of 0.5 µg *vif* as well as 1 µg HIV-1-GFP (Δenv, Δvif, Δvpr, Δnef) and 0.5 µg p-L-VSV-G. Virus was collected 24 h later, and then added to Jurkat T cells. Virus transfer was standardized across treatment conditions by p24 levels as described (Materials and Methods). Percent infected cells was then determined using FACS analysis for GFP-expression after virus was allowed to incubate with target cells for 72 hours. Error bars represent one standard deviation.

In order to identify proteins that interact with APOBEC3G, we performed a mass spectrometry analysis of proteins co-immunoprecipitated with APOBEC3G from primary CD4^+^ T cells or 293T cells. We found that the putative RNA helicase, Mov10, was reproducibly and predominantly associated with high-molecular weight APOBEC3G preparations. This finding was in line with observations from other reports that detected Mov10 in similar mass spectrometry analysis [6], [9]. Although in subsequent analysis we did not find a direct association between APOBEC3G and Mov10 (data not shown), we asked whether perturbation of Mov10 expression could impact HIV-1 infectivity in a manner similar to APOBEC3G. Accordingly, 293T cells were transfected with different Mov10 plasmid amounts. Mov10 was highly expressed upon transfection (Fig. 1A), and this expression level was not immediately toxic to cells as no cell death was observed over three days following transfection (data not shown).

We next determined whether viruses produced in the presence of ectopic Mov10 exhibited reduced infectivity similar to that typically observed with APOBEC3G-transfected 293T cells. To address this point, we co-transfected 293T cells with GFP-expressing HIV-1 (with or without Vif), a vesicular somatitis virus glycoprotein (VSV-G) expression construct, and either a Mov10 or APOBEC3G expression plasmid. Two days later, VSV-G pseudotyped HIV-1 vectors produced from the transfected 293T cells were used to infect the Jurkat T cell line. Remarkably, we found that overexpression of Mov10 almost completely abolished infectivity of HIV-1 produced by 293T cells (Fig. 1B). However, in contrast to APOBEC3G overexpression, the Mov10-mediated reduction of HIV-1 infectivity could not be rescued by co-expression of HIV-1 *vif* (Fig. 1B). These data suggested that increased levels of Mov10 have a profound effect on the generation of infectious HIV-1.

**Figure 2:**
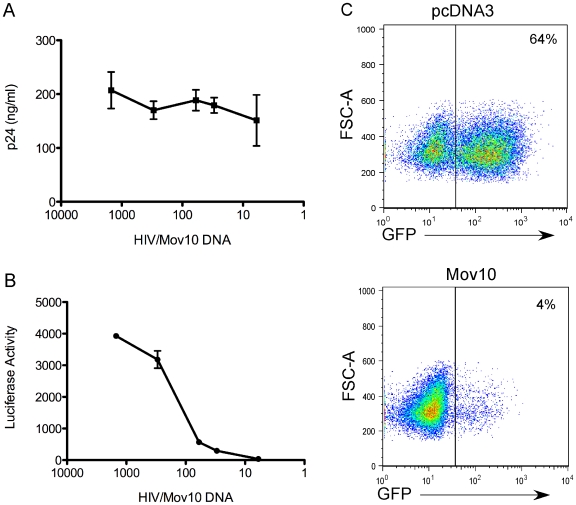
Mov10 decreases specific infectivity of HIV-1. Supernatants of 293T cells that had been transfected with varying amounts of Mov10-expressing plasmid were assayed for (**A**) HIV-1.Luc p24 levels and (**B**) after standardization by p24 content, infectivity of target cells was determined by luciferase activity from VSVG.HIV.Luc, which encodes all the HIV accessory genes. For simplicity, the amount of Mov10 plasmid that was transfected is expressed as a ratio of HIV-1 plasmid to Mov10 plasmid and plotted logarithmically. In the experiment (see Materials and Methods for further explanation) HIV-1 plasmid levels remained constant, while Mov10 plasmid was used at levels of 1/6 to 1/1500 that of the HIV-1 plasmid. Error bars represent one standard deviation. (**C**) Representative plots of Jurkat T cells infected with GFP-expressing virus produced in the presence of either pcDNA3 or Mov10 (ratio of Mov10 to HIV-1 plasmid was 1/25) three days after infection. Quantification of experiments performed using GFP-expressing virus is shown in supplemental figure 1.

We then asked whether the suppression of HIV-1 infectivity through Mov10 overexpression was due to reduced HIV-1 particle production. We found that at varying ratios of plasmids encoding HIV-1 to Mov10, the HIV-1 particle production, as assessed by p24 protein levels in supernatant, was mostly unaffected (Fig. 2A and Fig. S1A); while the infectivity of the viruses normalized to p24 levels were greatly reduced using either luciferase- or GFP-expressing viruses (Fig. 2B, 2C and Fig. S1B). However, at the ratios where Mov10 levels were highest, we also observed a notable reduction in the level of p24 generated by producer cells (Fig. 2A and Fig. S1A, lowest HIV-1/Mov10 expressing plasmid ratios). Similar inhibition of HIV-1 infectivity was observed when viruses were produced from 293T cells stably overexpressing Mov10 for prolonged periods (Fig. S2).

**Figure 3:**
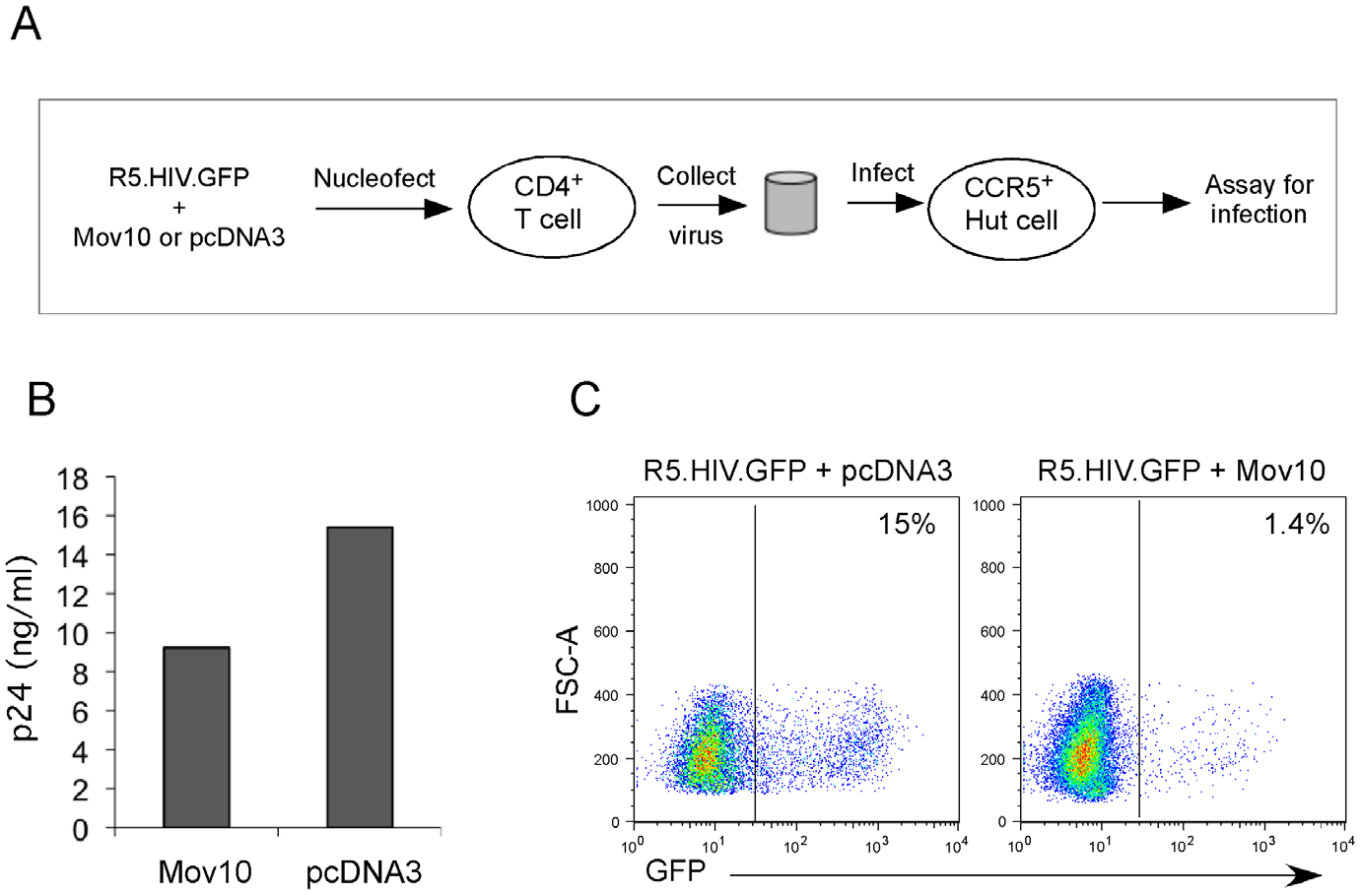
Mov10 impairs HIV-1 replication in primary CD4+ T cells. Supernatants of activated CD4^+^ cells that had been nucleofected with a replication competent, CCR5-tropic HIV-1 viral plasmid and either Mov10 or control (pcDNA3) plasmid ([**A**] schematic) were assayed for p24 production (**B**) and then standardized by p24 concentration and used to infect CCR5^+^ Hut cells. (**C**) Infection success was determined by flow cytometry analysis of GFP expression.

**Figure 4:**
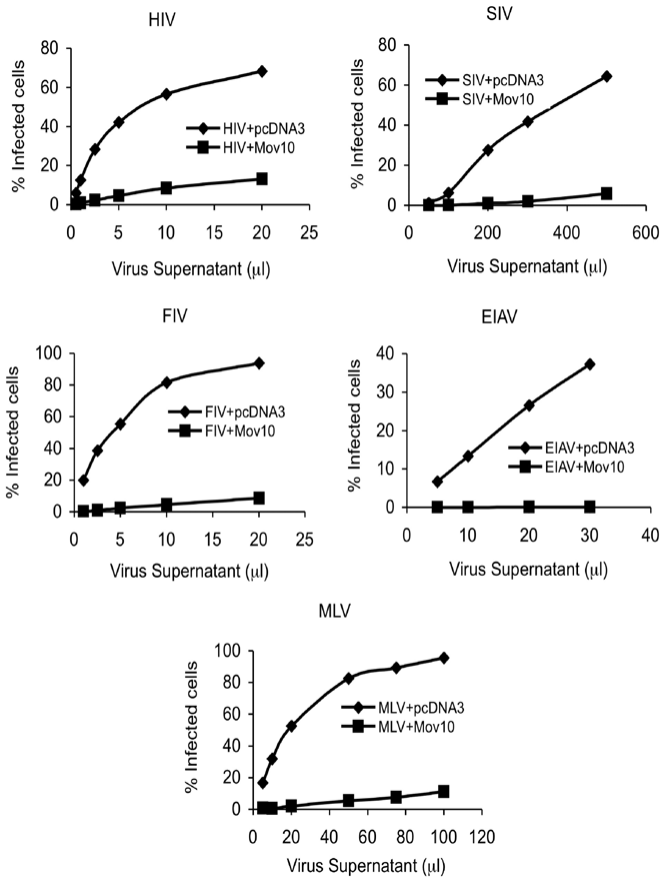
Broad inhibition of infectious retroviruses by Mov10. Virions derived from 293T cells transfected with various viral plasmids (as described in Materials and Methods) and either pcDNA3 or pcDNA3-Mov10 were used to infect HeLa cells. The % infected cells represents the percentage of GFP-positive cells in the cell population.

We next determined whether Mov10 could also inhibit the generation of replication-competent HIV-1 from primary human CD4^+^ T cells. For this experiment, highly purified CD4^+^ T cells were nucleofected with a replication-competent, CCR5-tropic HIV-1 plasmid (R5.HIV.GFP) in the presence of a Mov10 expression plasmid or control plasmid (pcDNA3). The virus produced from CD4^+^ T cells from this transfection was in turn used to infect CCR5^+^ Hut78 T cells (experimental setup shown in Fig. 3A). Virus production in primary cells was slightly impaired in the presence of Mov10 (Fig. 3B). However, when supernatants containing identical levels of p24 were applied to Hut78 cells, a significant reduction in HIV-1 infectivity due to Mov10 overexpression was observed (Fig. 3C). Next, we co-transfected viral plasmids for other retroviruses, SIV, MLV, FIV and EIAV, along with the Mov10 expression plasmid or empty vector to produce the respective viruses in presence or absence of Mov10. We tested their infectivity on HeLa cells and found that in the presence of Mov10, similar to HIV-1, infectivity of all of the tested lentiviruses and the retrovirus were profoundly suppressed (Fig. 4).

**Figure 5:**
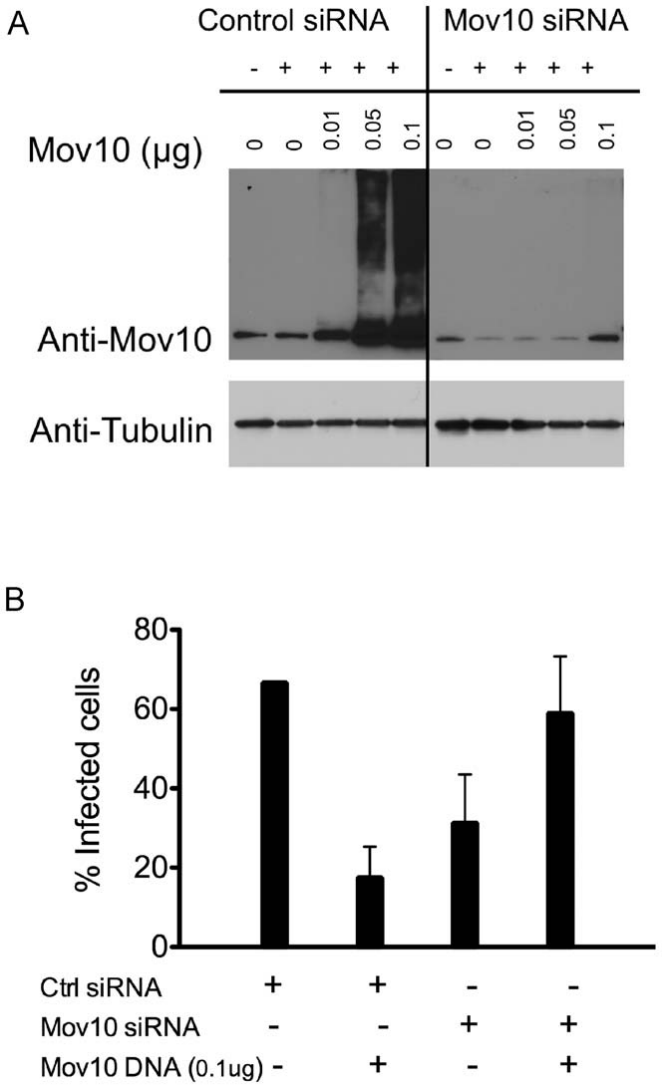
An optimal concentration of Mov10 is required for HIV-1 infectivity. 293T cells were transfected with a non-targeting siRNA or Mov10-specific siRNA. At 48 h post-siRNA transfection, the cells were transfected with 1.5 µg of pHIV-RFP, 0.7 µg of p-L-VSV-G and increasing amounts of the Mov10 expression plasmid. At 96 h post-siRNA transfection: (**A**) the cell lysates were examined by Western blot for Mov10 levels using an anti-Mov10 antibody. Equal protein loading was confirmed by probing with anti-tubulin antibody. (**B**) After normalizing for p24 values, virus obtained from the transfections was used to infect HeLa cells and infectivity was measured by FACS. The % infected cells represents the percentage of RFP-positive cells in the cell population. Error bars represent one standard deviation.

Because overexpression of Mov10 impaired HIV-1 infectivity, we asked whether endogenously expressed Mov10 was also inhibitory to HIV-1 replication. To address this we silenced Mov10 expression in producer cells using siRNAs targeting Mov10. Surprisingly, reduced levels of Mov10 also suppressed HIV-infectivity (Figure 5), suggesting a positive role for Mov10 in the generation of optimally infectious virions. To further test whether Mov10 levels were indeed critical or whether the siRNA was acting “off target,” Mov10 was ectopically expressed in siRNA-treated cells from which HIV-1 was produced. Because high levels of Mov10 would be inhibitory to HIV-1, the knockdown cells were complemented with a wild-type form of Mov10 that remained susceptible to the siRNA pool to restrict increased expression. In the absence of Mov10 siRNA, transfection of increasing amounts of the Mov10 expression plasmid leads to a dramatic increase of Mov10 levels even at relatively low input DNA amounts (Fig. 5A, left). At the highest levels of Mov10 (0.1 µg DNA), the specific infectivity of HIV-1 is diminished (Fig. 5B). In the presence of Mov10 siRNA, Mov10 levels, relative to protein loading controls, are less dramatically increased by Mov10 plasmid transfection (Fig. 5A, right). Without Mov10 plasmid transfection, siRNA targeting of endogenous Mov10 led to a 2-fold reduction in HIV-1 particle infectivity (Fig. 5B). The restoration of Mov10 expression to endogenous levels (0.1 µg DNA) was sufficient to increase HIV-1 specific infectivity. These data indicate that endogenous Mov10 aids in HIV-1 replication and that slight variation from the wild type level of Mov10 can drastically affect the infectivity of HIV-1.

### How Does Mov10 Decrease Infectivity of HIV-1

**Figure 6:**
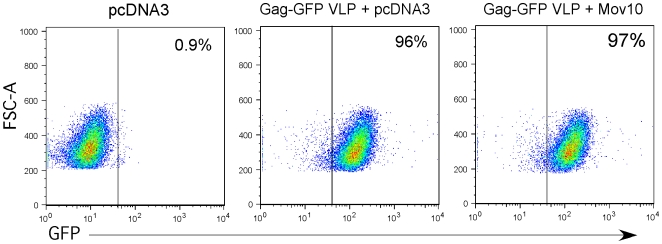
Viral glycoprotein incorporation is not affected by Mov10 overexpression. 293T cells were transfected with plasmids necessary for the production of VSV-G pseudotyped Virion-Like Particles (VLPs) containing a GFP tag (as a Gag-GFP fusion) as well as either control or Mov10. Supernatant from these cells was then collected and added to Jurkat T cells and assayed for binding to target cells. Cells bound by one or more VLPs are GFP^+^ by FACS.

**Figure 7:**
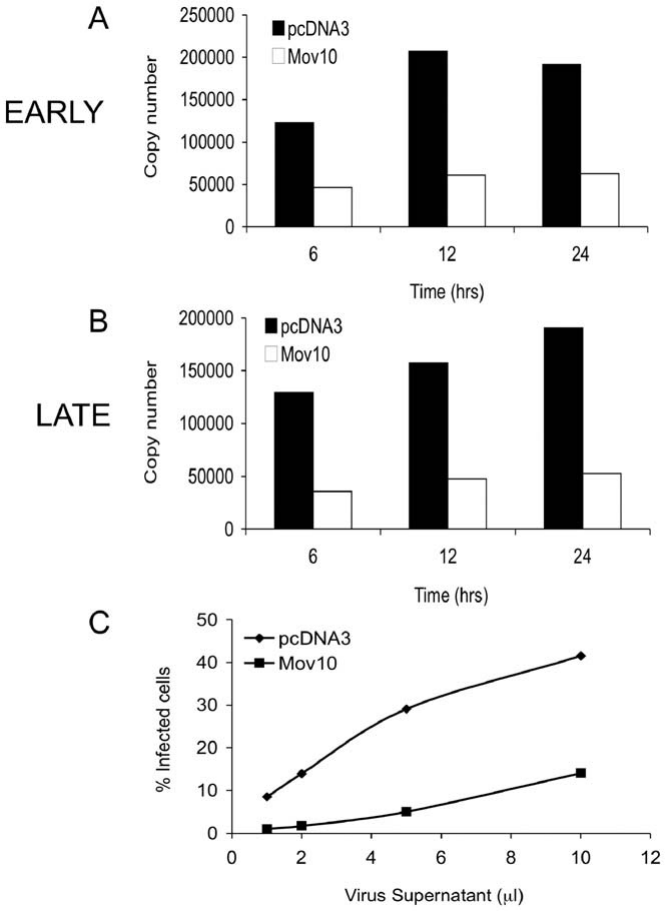
Early and late reverse transcription is suppressed in virus produced from cells overexpressing Mov10. Synthesis of reverse transcripts by real-time PCR after infection by HIV-1 produced in the presence of either empty vector (pcDNA3) or Mov10 expressing plasmid was measured. No significant difference between production of (**A**) Early (R-U5) or (**B**) Late (R-Gag) DNA products of reverse transcription was observed. (**C**) Infectivity of virions produced in the presence of Mov10 was significantly inhibited. Input viruses were normalized for p24. Results are representative of one out of three similar experiments.

In order to determine how Mov10 reduces the infectivity of HIV-1, we analyzed the early events in the lifecycle of HIV-1 produced in the presence of perturbed Mov10 levels. Accordingly, we first determined whether Mov10 interfered with the incorporation of viral glycoproteins in HIV-1 particles. We constructed virion-like particles (VLP) that express GFP through fusion to Gag and trans-incorporate VSV-G. The VLPs were then used to assess their binding capacity to a T cell line by analysis for GFP. When the target cells were analyzed by flow cytometry, there was no decrease in the ability of VLPs produced in the presence of Mov10 to bind to Jurkat cells (Fig. 6). We next asked whether Mov10-treated HIV-1 has defects during its early, post-entry stages. We used quantitative real-time PCR to analyze the early and late HIV-1 reverse transcripts (Fig. 7). Compared to virus from cells expressing a control plasmid, virus from Mov10-overexpressing cells was 80% less efficient in synthesis of early reverse transcripts (minus-strand strong-stop DNA). This suggests that the defect induced by Mov10 manifests prior to the initiation of reverse transcription during the early stages of post-entry replication of the virus.

### Mov10's Anti-HIV-1 Activity Is Independent of Its Putative RNA Helicase Domain

**Figure 8:**
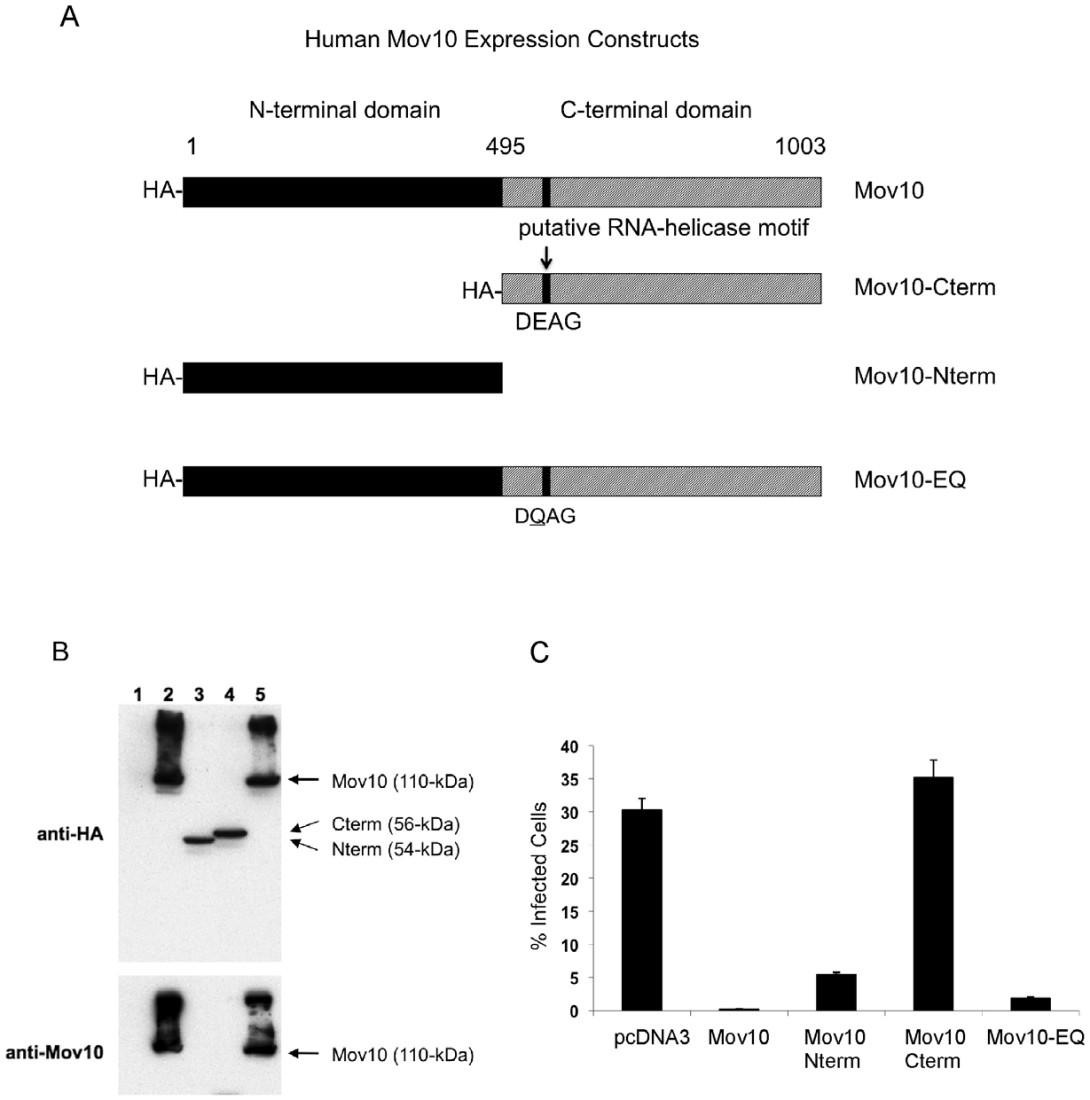
The helicase domain of Mov10 is not required for HIV-1 restriction. Virions were produced by cotransfection of 293T cells with HIV-1 vector and either empty vector or Mov10, Mov10 N-terminus, Mov10 C-terminus or the putative helicase motif mutant expression plasmids. (**A**) Schematic of wild-type and mutated human Mov10 constructs. (**B**) Cell lysates were probed with anti-HA, anti-Mov10, and anti-tubulin. Lanes: 1) pcDNA3, 2) Mov10, 3) Mov10 Nterm, 4) Mov10 Cterm, and 5) Mov10-EQ. Arrows indicate molecular weight of native Mov10. (**C**) Infectivity of virions produced was examined by FACS after infection of HeLa cells. The % infectivity represents the percentage of RFP-positive cells in the cell population. Error bars represent one standard deviation.

Because a potential RNA helicase domain has been identified for Mov10 and because RNA helicases have been implicated as modulators of HIV-1 replication [13], [14], we hypothesized that Mov10's antiviral activity could be due to the presence of its putative helicase domain. To analyze the roles of the particular domains of Mov10, we split the protein into N-terminal and C-terminal portions (Fig. 8A). The C-terminal half of the protein contains the putative RNA helicase domain of the protein [11] while the N-terminal half is not yet characterized [15], [16]. In addition we generated a point mutation of a motif predicted to be essential for ATP binding to the potential helicase (Fig. 8A), based upon previous reports of an inactivating mutation in an RNA helicase [17]. HIV-1 particles were produced by co-transfection of 293T cells in the presence of either empty vector, Mov10, Mov10 N-terminus, Mov10 C-terminus or the helicase domain mutant of Mov10. All HA-tagged proteins were detectably expressed in cells (Fig. 8B). Upon examining the infectivity of the produced virions, we found that both the N-terminal half of Mov10, which lacks the helicase domain, and the helicase domain point mutant diminished infectivity of HIV-1 particles nearly as well as wild type Mov10 (Fig. 8C). By contrast, the C-terminal domain alone had no effect. These data show that the N-terminal portion of the protein was required for Mov10-mediated virus suppression and that the putative RNA helicase domain did not contribute to Mov10's antiviral activity under these experimental conditions.

## Discussion

In this study, we have shown that the RNA helicase Mov10 can modulate the production of infectious HIV-1. Ectopic expression of Mov10 diminishes per-particle infectivity resulting in virus impaired at an early step of infection in target cells. This effect is observed in both primary and transformed cells using HIV-1 single-cycle vectors or replication-competent genomes. Importantly, endogenous Mov10 appears to contribute to HIV-1 replication as virions produced from cells that have been depleted of Mov10 are also less infectious. These data suggest that Mov10 is at a critical nexus of HIV-1 replication and perturbation of this factor is restrictive to the virus. Notably, other retroviruses are also sensitive to elevated expression of Mov10.

Mov10 has been found in association with Ago1 and Ago2 in the RISC, together with TNRC6B, which are also found to localize to P-bodies [8], [18], [19]. In addition, ectopically expressed Mov10 appears to be enriched in P-bodies [20], similar to APOBEC3G [6], [21]. While P-body components are essential for retrotransposition of yeast Ty1 and Ty3 elements [22], [23], their role in retroviral replication has not been established. Perturbation of P-bodies could affect cellular RNA metabolism and thus limit HIV-1 production. However, we observed no quantitative change in levels of intracellular HIV-1 RNA (data not shown), particle release, or vRNA incorporation in particles (data not shown). The infectivity defect was apparent after normalizing for particle amounts. Unlike GW182 or other P-body components, Mov10 is not known to be essential to the genesis or turnover of P-bodies. These data suggest that the role of Mov10 in HIV-1 replication could be independent of RNA metabolism.

The incorporation of APOBEC3G by different retroviruses suggests that they may traffic via P-bodies during viral production. Indeed, Mov10 was also reported to be incorporated in HIV-1 particles [24]. The presence of a RISC component in HIV-1 particles is provocative given that RISC may be physically associated with multivesicular bodies [25], which are essential for the production and release of retroviruses [2]. It is tempting to speculate that retroviral RNA modifications or association with the viral core components may require RISC or P-body machinery, which in turn could be disrupted by modulating Mov10 expression levels. Understanding how Mov10 interacts with HIV-1 and cellular machinery may eventually help reveal its mechanism of inhibition when its expression is perturbed.

Paradoxically, silencing a portion of Mov10 expression in producer cells also reduced infectivity of HIV-1, albeit with much lower potency compared to inhibition seen in over-expression experiments. It will be of interest to determine whether the removal of Mov10 from virions or the absence of functional Mov10 in virus-producers cells underlies the loss in infectivity. Alternatively, it is conceivable that perturbation of Mov10 levels disrupts other components of the P-body machinery, which are required for viral RNA processing and assembly. It will also be important to determine the physiological function of Mov10 in primary human T cells and macrophages, which are the natural targets of HIV-1.

Mov10 may prove to be an attractive therapeutic target given the small window in which cellular levels are compatible with production of infectious HIV-1. Elevated levels of Mov10 in primary cells are sufficient to hinder HIV-1 replication, and it is conceivable that pharmacological treatment of these cells with drugs that stabilize or increase P-body numbers may in turn increase Mov10 levels. Notably, Mov10 lacking an RNA interaction domain through limited mutagenesis or complete removal of the C-terminal domain is antiviral when ectopically expressed. Thus fragments of the protein could be used as antivirals and potentially minimize effects on the cell biology. The N-terminal domain also provides an attractive tool with which to select for HIV-1 resistance to better understand the interaction of Mov10 with the virus. However, it is not yet clear how the N-terminal half of Mov10, which lacks the putative helicase domain, is potently functional in reducing HIV-1 infectivity. It is possible that the N-terminal domain, could either be interacting with and disrupting other components of the P-body machinery, or it may have a yet to be identified function in RNA processing.

The effect of Mov10 depletion or overexpression on HIV-1 replication is reminiscent of the effect that the ESCRT component Tsg101 can have on HIV-1 production [26]. Depletion of Tsg101 impairs HIV-1 release, overexpression of Tsg101 interferes with HIV-1 release, and expression of Tsg101 fragments prevents HIV-1 Gag interactions with Tsg101 or other ESCRT components thus blocking virus release. As our knowledge of cellular proteins manipulated by HIV-1 expands, we expect that more proteins will be discovered that exhibit similar characteristics to Mov10—these being a narrow expression window in which HIV-1 is capable of reproducing. HIV-1's requirement of an optimal level of Mov10 during virion production is reminiscent of Robert Southey's classic tale of Goldilocks seeking nourishment and comfort that comported to a narrow range when sojourning in an ursine abode [27]. Proteins with such Goldilocks quality provide excellent, potential targets for therapy because perturbation of their levels in either direction reduces HIV-1's ability to reproduce.

## Materials and Methods

### Cell Purifications and Culture

Blood samples were obtained from anonymous healthy donors as buffy coats (New York Blood Center). New York Blood Center obtains written informed consent from all participants involved in the study. Because all the samples were sent as anonymous, the Institutional Review Board at New York University medical center determined that our study was exempt from further ethics approval requirement. Peripheral Blood Mononuclear cells (PBMC) were isolated with Ficoll-Hypaque (Amersham Pharmacia). CD4^+^ T cells were isolated from PBMC using magnetic bead sorting (Invitrogen, Dynabeads). Purified CD4^+^ T cells were activated using anti-CD3/CD28 coated beads (Dynabeads, Invitrogen) and cultured in RPMI media (Life Technologies) with 10% fetal calf serum (FCS; Atlanta Biologicals) and supplemented with IL-2 (200 U/ml). Jurkat and Hut78 cells were also grown in RPMI-10% FCS media. HEK293T and HeLa cell lines were maintained in DMEM supplemented with 10% FCS, 100 U/ml penicillin and 0.1 mg/ml streptomycin.

### Plasmids and Mov10 Mutants

The HIV-RFP plasmid was constructed from the HIV-EGFP plasmid [28] and has been described previously (Lee et al., in press). HIV Vif gene cloned in pcDNA 3 [29] was obtained from NIH AIDS reagent repository. Wild type Mov10 (NCBI accession number BC009312.2, cDNA obtained from Origene) and mutant Mov10s, Mov10-Nterm (corresponds to amino acids 1–495), Mov10-Cterm (corresponds to amino acids 496–1003) and Mov10.EQ (putative helicase motif mutant) were PCR amplified and subcloned into the pcDNA3 vector that, contained an in frame 5′ HA tag, using BamHI and NotI restriction sites to create HA-tagged mutants. Following primers were used for PCR amplifications: For full-length Mov10: 5′ TAC GCC GGA TCC CCC AGT AAG TTC AGC TGC CGG CAG – 3′ CGT TAG GCG GCC GCT CAG AGC TCA TTC CTC CAC TCT GGC TCC. For Nterm mutant: 5′ TAC GCC GGA TCC CCC AGT AAG TTC AGC TGC CGG CAG – 3′ CGT TAG GCG GCC GCT CAC CGG TCG TAC AGC TTG AGT TTC ACA. For Cterm Mov10: 5′TAC GCC GGA TCC AGT CTG GAG TCA AAC CCA GAG CAG, 3′ CGT TAG GCG GCC GCT CAG AGC TCA TTC CTC CAC TCT GGC TCC. The point mutation in the DEAG sequence of Mov10 was created by replacing glutamate residue at position 646 with glutamine (DEAG to DQAG) and named Mov10-EQ. APOBEC3G was similarly subcloned into pcDNA3 vector for transfections. The pNL4.3.R-.E-.Luc vector was kindly provided by Dr. Nathaniel Landau (New York University). Replication competent CCR5-tropic HIV (R5.HIV) expressing GFP was previously described [30]. Virion-like particles (VLPs) were generated using plasmid expressing HIV-1 Gag fused to GFP (kind gift of Dr. Paul Spearman, Emory University).

### Virus Stocks

Virus stocks were produced by DNA transfection on monolayer cultures of 293T cells grown in six-well plates (Corning) using either Hilymax (Dojindo) or Lipofectamine 2000 (Invitrogen) transfection reagents. To produce HIV-1 vectors (VSVG.HIV-RFP or VSVG.NL4.3.R-.E-.Luc), each well of 293T cells in a six-well plate was cotransfected with 3 µg of HIV and 0.5 µg of p-L-VSV-G [31]. In experiments where Mov10 was also cotransfected, the plasmid was used in amounts ranging from 0.5 µg to 0.002 µg, which corresponds to ratios of HIV-1/Mov10 of 1/6 to 1/1500. 293T cells were cotransfected with 2.5 µg of pMIGR1 [32], 1.5 µg of pJK3 [31], 0.5 µg of pCMV-Tat and 1 µg of p-L-VSV-G plasmids [31] to produce the murine leukemia virus (MLV) stock; with 2 µg of pV1EGFP (SIV vector) and 2 µg of pUpSVOΔψ (SIV structural proteins) (kindly provided by Hung Fan) and 0.5 µg of pCMV-VSVG to produce the simian immunodeficiency virus (SIV) stock; with 2 µg of pGinSin (FIV vector) and 2 µg of pFP93 (FIV structural proteins) (kindly provided by Eric Poeschla) and 0.5 µg CMV-VSVG to produce the feline immunodeficiency virus (FIV) stock; and with 2 µg of p6.1G3CeGFPW (EIAV vector), 2 µg of pEV53B (EIAV structural proteins) (kindly provided by John Olsen) and 0.5 µg of pCMV-VSV-G to produce the equine infectious anemia virus (EIAV) stocks. To examine the effects of Mov10 on viruses produced, the 293T cells were also cotransfected with either the empty pcDNA3 plasmid as control or the pcDNA3-HA-Mov10 plasmid. Culture supernatants from the 293T cells were collected 48 h post-transfection, clarified by low-speed centrifugation (1,000×*g*, 10 min), and filtered through 0.45 µm pore-size sterile filters.

### HIV-1 p24 ELISA

For the HIV-1 vectors, the clarified supernatants were analyzed for p24 antigen concentration by enzyme-linked immunosorbent assay (PerkinElmer) following manufacturer's instructions. HRP levels were detected via colorimetry and quantified following manufacturer's protocol on an Envision 96-well plate reader (PerkinElmer). HIV-1 capsid monoclonal antibody was obtained through the NIH AIDS Research and Reference Reagent Program, Division of AIDS, NIAID, NIH (183-H12-5C, contributed by Bruce Chesebro and Hardy Chen). Secondary antibodies included HRP-conjugated goat anti-mouse, goat anti-human, and goat anti-rabbit antibodies (GE Healthcare).

### Infection Assays

Infectivities of non-HIV retroviruses were determined either by titration of virus supernatants on HeLa cells and of HIV-1 on Jurkat or Hut78 cells using virus samples normalized by p24 (capsid) levels. The expression of GFP or RFP following infection by the HIV-1, MLV, SIV, FIV and EIAV viruses was measured by fluorescence-activated cell sorter (FACS) analysis (FACSCalibur, Becton Dickinson). The percent infected cells represents the percentage of GFP-positive or RFP-positive cells in the cell population. Alternatively, for luciferase reporter viruses, Jurkat cells (1.5×10^4^ per well) were infected for 3 days with VSV-G-pseudotyped NL4.3.R-.E-.Luc (VSVG.HIV.Luc) and luciferase activity was measured by LucLite kit from PerkinElmer according to the manufacturer's protocol using an Envision 96-well plate reader (PerkinElmer).

### Knockdown of Endogenous Mov10

Transient silencing of target genes was achieved by transfecting the gene-specific siRNAs (Dharmacon, ON-TARGETplus SMARTpool L-014162-00) into 293T cells using Oligofectamine (Invitrogen). 50 nM of the nontargeting control siRNA or gene-specific siRNA was transfected into 293T cells 48 h prior to plasmid transfections. Knockdown of Mov10 protein was confirmed by Western blot.

### Nucleofection of Primary T Cells

One day after activation, sorted human CD4^+^ were nucleofected using the Amaxa Nucleofector System (Lonza) with R5.HIV.GFP plasmid (1 µg) and either Mov10 or pcDNA3 plasmid (0.5 µg). 48 hours after nucleofection, viral supernatants were collected and analyzed for p24 concentration by ELISA as previously described [30]. Aliquots containing 100 pg of p24 were then used to infect 1.5×10^4^ CCR5^+^ Hut78 cells. After 3 days of culture percent of infected cells was analyzed by FACS.

### Real-Time PCR Analysis of Virus Infection

The 293T-derived HIV-1 virions were treated with 50 U/ml DNaseI (Roche) for 30 minutes. These DNaseI treated HIV-1 virions were standardized by p24 concentration and then used to infect HeLa cells for different times. At each time points cells were collected, lysed and total DNA was extracted using the QIAmp Blood Mini Kit (Qiagen). As a control for plasmid contamination, an equivalent amount of virus was either boiled for 10 minutes before infecting cells or the reverse transcriptase inhibitor efavirenz (100 nM) was added at the time of infection. Samples were assayed by Real-Time PCR using Platinum qPCR SuperMix-UDG (Invitrogen) with primers, probes and PCR conditions as described previously [33]. Duplicate samples of serial dilutions of plasmid DNAs containing the target sequences were used to generate a standard curve, which was used for quantification of PCR products.

### Western Blot Analysis

Transfected cells were lysed and solubilized in RIPA buffer (Sigma) (50 mM Tris-HCl, pH 8.0, with 150 mM sodium chloride, 1.0% Igepal CA-630 [NP-40], 0.5% sodium deoxycholate, and 0.1% sodium dodecyl sulfate). The cell lysates were then mixed with a 2X Laemmli sample loading buffer (BioRad) (62.5 mM Tris-HCl, pH 6.8, 25% glycerol, 2% SDS, 0.01% bromophenol blue and 5% 2-mercaptoethanol), boiled and then samples were loaded and separated on 10.0% polyacrylamide gels containing SDS. Following electrophoresis, proteins were transferred to a PVDF membrane by electroblotting and incubated for 1 hr at room temperature in blocking buffer (5% nonfat dry milk in PBS). The blocked blot was exposed to the appropriate primary antibody in blocking buffer with constant mixing. After extensive washing, bound antibodies were detected by chemiluminescence using horseradish peroxidase-conjugated, species-specific, secondary antibodies as described by the manufacturer (GE Healthcare). The following antibodies were used for the western blot analysis: anti-Mov10 (Proteintech Group, Inc.), anti-HA epitope (Sigma) and anti-α-tubulin (Sigma), using the manufacturer's recommended antibody concentrations.

## Supporting Information

Figure S1 Mov10 decreases specific infectivity of HIV-1. Supernatants of 293T cells that had been transfected with varying amounts of Mov10-expressing plasmid were assayed for (A) HIV-1 CA (p24) levels and (B) infectivity after standardization by p24 content. The particular HIV-1 vector used in this experiment expresses GFP and lacks any of the HIV accessory genes (Vif, Vpr, Nef and Vpu). For simplicity, the amount of Mov10 plasmid that was transfected is expressed as a ratio of HIV-1 plasmid to Mov10 plasmid and plotted logarithmically. In the experiment (see “Materials and Methods” for further details) HIV-1 plasmid levels remained constant, while Mov10 plasmid was used at levels of 1/6 to 1/1500 that of the HIV-1 plasmid. Error bars represent one standard deviation.
(0.33 MB TIF)

Figure S2 HIV-1 produced in cells stably expressing Mov10 is less infectious. 293T cells were stably transfected with a vector expressing Mov10 and selected with 0.5 mg/ml G418. When these cells were subsequently transfected with an HIV-1 vector, the virus produced from them was less infectious to Jurkat T cells than virus produced in cells stably transfected with a control (pcDNA3) vector. Error bars represent standard error of the mean.
(0.24 MB TIF)

## References

[1] Bushman FD, Malani N, Fernandes J, D'Orso I, Cagney G (2009). Host cell factors in HIV replication: meta-analysis of genome-wide studies.. PLoS Pathog.

[2] Bieniasz PD (2009). The cell biology of HIV-1 virion genesis.. Cell Host Microbe.

[3] Strebel K, Luban J, Jeang KT (2009). Human cellular restriction factors that target HIV-1 replication.. BMC Med.

[4] Cullen BR (2006). Role and mechanism of action of the APOBEC3 family of antiretroviral resistance factors.. J Virol.

[5] Sheehy AM, Gaddis NC, Choi JD, Malim MH (2002). Isolation of a human gene that inhibits HIV-1 infection and is suppressed by the viral Vif protein.. Nature.

[6] Gallois-Montbrun S, Kramer B, Swanson CM, Byers H, Lynham S (2007). Antiviral protein APOBEC3G localizes to ribonucleoprotein complexes found in P bodies and stress granules.. J Virol.

[7] Beckham CJ, Parker R (2008). P bodies, stress granules, and viral life cycles.. Cell Host Microbe.

[8] Eulalio A, Behm-Ansmant I, Izaurralde E (2007). P bodies: at the crossroads of post-transcriptional pathways.. Nat Rev Mol Cell Biol.

[9] Kozak SL, Marin M, Rose KM, Bystrom C, Kabat D (2006). The anti-HIV-1 editing enzyme APOBEC3G binds HIV-1 RNA and messenger RNAs that shuttle between polysomes and stress granules.. J Biol Chem.

[10] Schnieke A, Stuhlmann H, Harbers K, Chumakov I, Jaenisch R (1983). Endogenous Moloney leukemia virus in nonviremic Mov substrains of mice carries defects in the proviral genome.. J Virol.

[11] Dalmay T, Horsefield R, Braunstein TH, Baulcombe DC (2001). SDE3 encodes an RNA helicase required for post-transcriptional gene silencing in Arabidopsis.. Embo J.

[12] Haussecker D, Cao D, Huang Y, Parameswaran P, Fire AZ (2008). Capped small RNAs and MOV10 in human hepatitis delta virus replication.. Nat Struct Mol Biol.

[13] Ma J, Rong L, Zhou Y, Roy BB, Lu J (2008). The requirement of the DEAD-box protein DDX24 for the packaging of human immunodeficiency virus type 1 RNA.. Virology.

[14] Zhou Y, Ma J, Bushan Roy B, Wu JY, Pan Q (2008). The packaging of human immunodeficiency virus type 1 RNA is restricted by overexpression of an RNA helicase DHX30.. Virology.

[15] Cordin O, Banroques J, Tanner NK, Linder P (2006). The DEAD-box protein family of RNA helicases.. Gene.

[16] de la Cruz J, Kressler D, Linder P (1999). Unwinding RNA in Saccharomyces cerevisiae: DEAD-box proteins and related families.. Trends Biochem Sci.

[17] Askjaer P, Rosendahl R, Kjems J (2000). Nuclear export of the DEAD box An3 protein by CRM1 is coupled to An3 helicase activity.. J Biol Chem.

[18] Chendrimada TP, Finn KJ, Ji X, Baillat D, Gregory RI (2007). MicroRNA silencing through RISC recruitment of eIF6.. Nature.

[19] Baillat D, Shiekhattar R (2009). Functional dissection of the human TNRC6 (GW182-related) family of proteins.. Mol Cell Biol.

[20] Meister G, Landthaler M, Peters L, Chen PY, Urlaub H (2005). Identification of novel argonaute-associated proteins.. Curr Biol.

[21] Niewiadomska AM, Tian C, Tan L, Wang T, Sarkis PT (2007). Differential inhibition of long interspersed element 1 by APOBEC3 does not correlate with high-molecular-mass-complex formation or P-body association.. J Virol.

[22] Checkley MA, Nagashima K, Lockett SJ, Nyswaner KM, Garfinkel DJ P-Body Components Are Required for Ty1 Retrotransposition during Assembly of Retrotransposition-Competent Virus-Like Particles.. Mol Cell Biol.

[23] Beliakova-Bethell N, Beckham C, Giddings TH, Winey M, Parker R (2006). Virus-like particles of the Ty3 retrotransposon assemble in association with P-body components.. Rna.

[24] Ott DE (2008). Cellular proteins detected in HIV-1.. Rev Med Virol.

[25] Gibbings DJ, Ciaudo C, Erhardt M, Voinnet O (2009). Multivesicular bodies associate with components of miRNA effector complexes and modulate miRNA activity.. Nat Cell Biol.

[26] Goila-Gaur R, Demirov DG, Orenstein JM, Ono A, Freed EO (2003). Defects in human immunodeficiency virus budding and endosomal sorting induced by TSG101 overexpression.. J Virol.

[27] Southey R (1837). The Story of The Three Bears..

[28] Unutmaz D, KewalRamani VN, Marmon S, Littman DR (1999). Cytokine signals are sufficient for HIV-1 infection of resting human T lymphocytes.. J Exp Med.

[29] Nguyen KL, llano M, Akari H, Miyagi E, Poeschla EM (2004). Codon optimization of the HIV-1 vpu and vif genes stabilizes their mRNA and allows for highly efficient Rev-independent expression.. Virology.

[30] Oswald-Richter K, Grill SM, Leelawong M, Tseng M, Kalams SA (2007). Identification of a CCR5-expressing T cell subset that is resistant to R5-tropic HIV infection.. PLoS Pathog.

[31] Bartz SR, Vodicka MA (1997). Production of high-titer human immunodeficiency virus type 1 pseudotyped with vesicular stomatitis virus glycoprotein.. Methods.

[32] Pear WS, Miller JP, Xu L, Pui JC, Soffer B (1998). Efficient and rapid induction of a chronic myelogenous leukemia-like myeloproliferative disease in mice receiving P210 bcr/abl-transduced bone marrow.. Blood.

[33] Julias JG, Ferris AL, Boyer PL, Hughes SH (2001). Replication of phenotypically mixed human immunodeficiency virus type 1 virions containing catalytically active and catalytically inactive reverse transcriptase.. J Virol.

