# Supplementary figures and images for: Perturbation of the P-Body Component Mov10 Inhibits HIV-1 Infectivity
Source: PLoS One. 2010 Feb 5;5(2):e9081. doi: 10.1371/journal.pone.0009081 (PMC2816699; doi:10.1371/journal.pone.0009081)

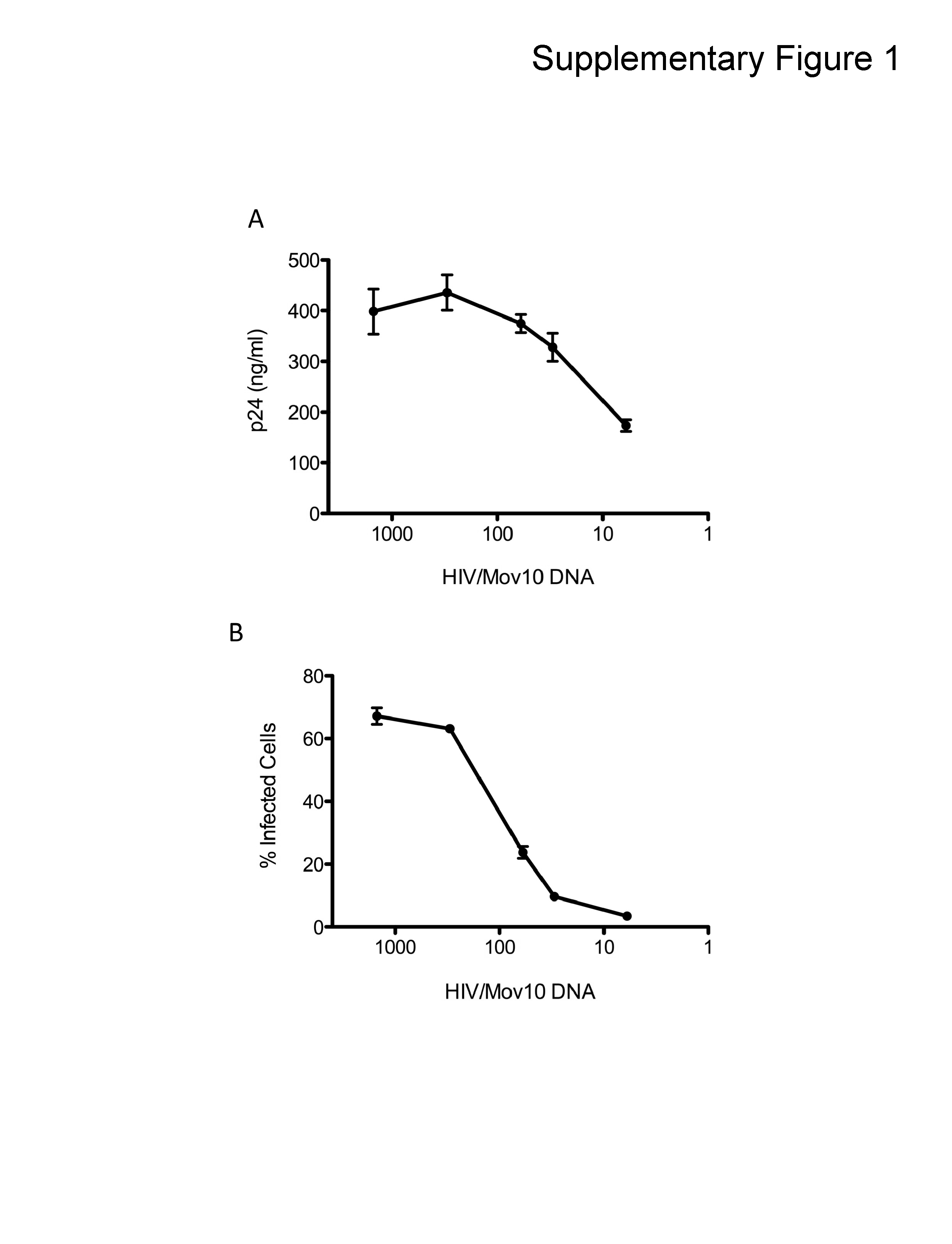

Supplement: Figure S1 — Mov10 decreases specific infectivity of HIV-1. Supernatants of 293T cells that had been transfected with varying amounts of Mov10-expressing plasmid were assayed for (A) HIV-1 CA (p24) levels and (B) infectivity after standardization by p24 content. The particular HIV-1 vector used in this experiment expresses GFP and lacks any of the HIV accessory genes (Vif, Vpr, Nef and Vpu). For simplicity, the amount of Mov10 plasmid that was transfected is expressed as a ratio of HIV-1 plasmid to Mov10 plasmid and plotted logarithmically. In the experiment (see “Materials and Methods” for further details) HIV-1 plasmid levels remained constant, while Mov10 plasmid was used at levels of 1/6 to 1/1500 that of the HIV-1 plasmid. Error bars represent one standard deviation. (0.33 MB TIF) [file pone.0009081.s001.tif]

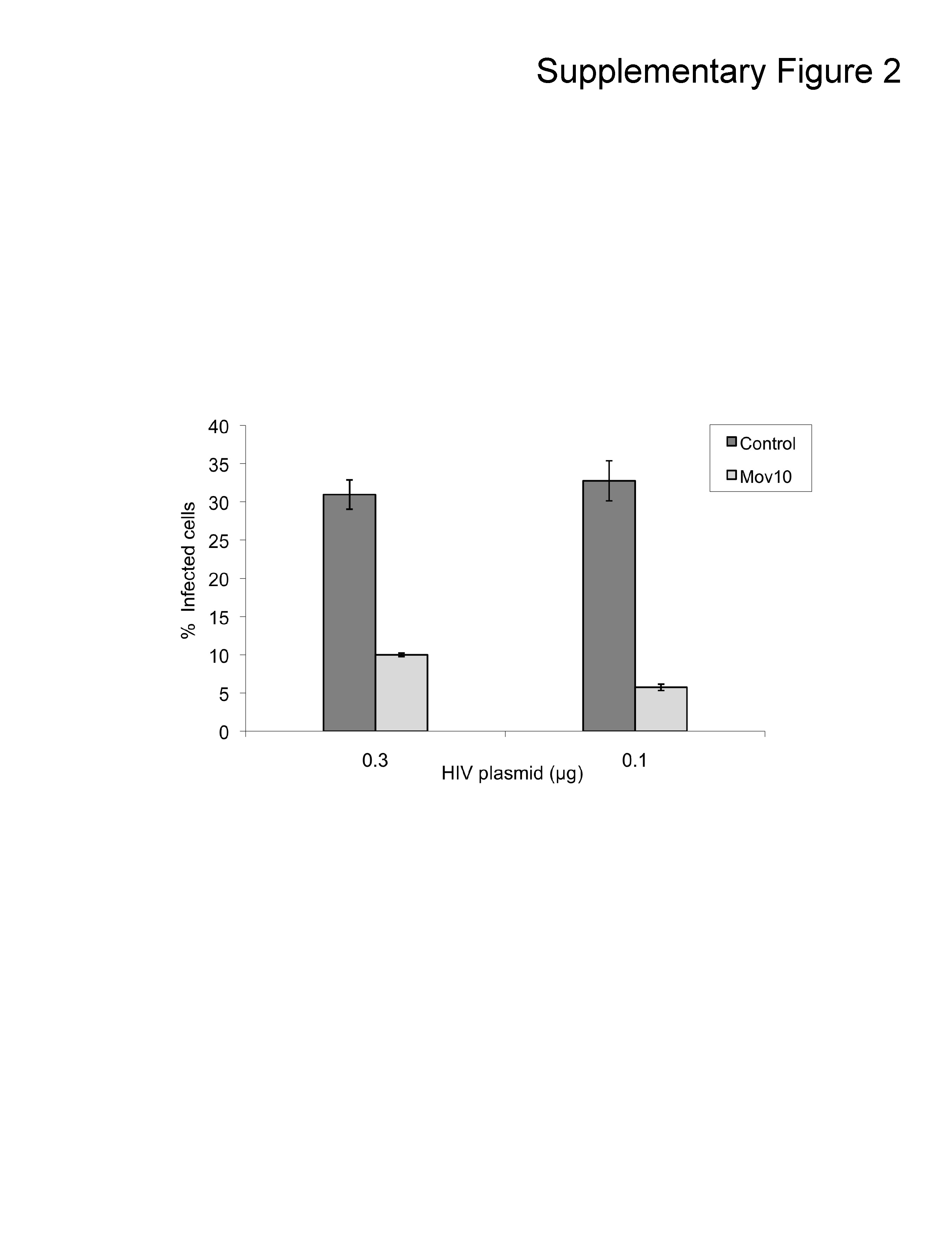

Supplement: Figure S2 — HIV-1 produced in cells stably expressing Mov10 is less infectious. 293T cells were stably transfected with a vector expressing Mov10 and selected with 0.5 mg/ml G418. When these cells were subsequently transfected with an HIV-1 vector, the virus produced from them was less infectious to Jurkat T cells than virus produced in cells stably transfected with a control (pcDNA3) vector. Error bars represent standard error of the mean. (0.24 MB TIF) [file pone.0009081.s002.tif]
